# *Candida auris*: A rapidly emerging cause of hospital-acquired multidrug-resistant fungal infections globally

**DOI:** 10.1371/journal.ppat.1006290

**Published:** 2017-05-18

**Authors:** Anuradha Chowdhary, Cheshta Sharma, Jacques F. Meis

**Affiliations:** 1 Department of Medical Mycology, Vallabhbhai Patel Chest Institute, University of Delhi, Delhi, India; 2 Department of Medical Microbiology and Infectious Diseases, Canisius-Wilhelmina Hospital, Nijmegen, the Netherlands; 3 Centre of Expertise in Mycology Radboudumc/CWZ, Nijmegen, the Netherlands; Geisel School of Medicine at Dartmouth, UNITED STATES

Candidiasis, which includes both superficial infections and invasive disease, is the most common cause of fungal infection worldwide. *Candida* bloodstream infections (BSI) cause significant mortality and elicit a major threat to intensive care unit (ICU) patients [1]. The annual global burden of *Candida* spp. BSIs is about 400,000 cases, with most cases reported from the developed world. Although *Candida albicans* remains the most frequently isolated *Candida* species in the clinical setting, in some countries, a marked shift towards species of *Candida* that have increased resistance to azoles such as fluconazole (FLU), the standard antifungal drug of choice in many countries, and to the recently introduced antifungals known as echinocandins, is reported. Several species of non-*albicans Candida*, such as *C*. *tropicalis*, *C*. *glabrata*, and *C*. *parapsilosis*, are well-recognized pathogens in BSIs in different geographic locations. More recently, *Candida auris*, a multidrug-resistant (MDR) yeast that exhibits resistance to FLU and markedly variable susceptibility to other azoles, amphotericin B (AMB), and echinocandins, has globally emerged as a nosocomial pathogen (Fig 1) [2–20]. Alarmingly, in a span of only 7 years, this yeast, which is difficult to treat and displays clonal inter- and intra-hospital transmission, has become widespread across several countries, causing a broad range of healthcare-associated invasive infections [4, 5, 10, 12, 21, 22].

**Fig 1:**
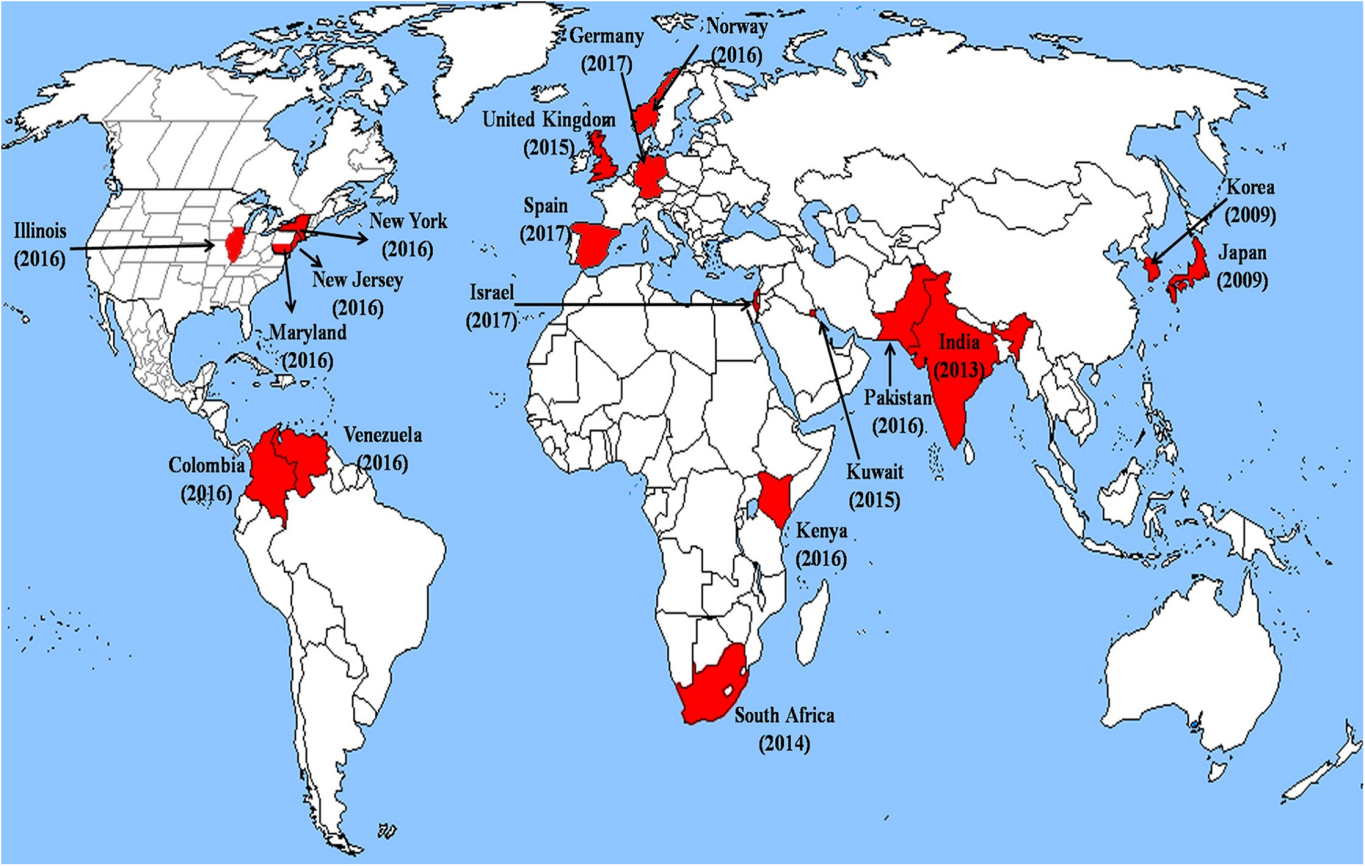
A global map depicting rapid emergence of multidrug-resistant clinical *Candida auris* strains in 5 continents. The value in parentheses denotes the year of report of *C*. *auris* from the respective country or state.

## Why is *C*. *auris* often misidentified in the routine microbiology laboratory?

In 2009, a novel *Candida* species, *C*. *auris*, in the *C*. *haemulonii* complex (Metchnikowiaceae), was first described from a patient in Japan after its isolation from the external ear canal [23]. The species exhibits a close phylogenetic relationship to *C*. *haemulonii* and is differentiated based on sequence analysis of the D1/D2 domain of the large ribosomal subunit (LSU) of 26S rRNA gene and the internal transcribed spacer (ITS) regions of the nuclear rRNA gene operon [23]. The first 3 cases of nosocomial fungemia due to *C*. *auris* reported in 2011 from South Korea highlighted the fact that this yeast is commonly misidentified as *C*. *haemulonii* and *Rhodotorula glutinis* by the commercial identification systems VITEK (BioMérieux, Marcy l’Etoile, France) and API-20C AUX (BioMérieux), respectively [3]. These systems involve precast panels of assimilation/growth tests using sets of carbon and nitrogen compounds and are still widely used for routine identification of yeasts. A comprehensive study from India investigated *C*. *auris* prevalence among 102 clinical isolates previously identified as *C*. *haemulonii* or *C*. *famata* with the VITEK system and found that 88.2% of the isolates were *C*. *auris*, as confirmed by ITS sequencing [9]. It is evident from several studies published recently that *C*. *auris* in routine microbiology laboratories remains an unnoticed pathogen, as 90% of the isolates characterized by commercial biochemical identification systems are misidentified primarily because of a lack of the yeast in their databases [3–9, 12, 16–19, 24, 25]. Different biochemical systems are used in microbiology laboratories, and the majority of them listed in Table 1 misidentify *C*. *auris*. A recent study on validating the identification of *C*. *auris* with 4 biochemical identification platforms found that all *C*. *auris* isolates were misidentified as *R*. *glutinis* by API-20C AUX, as *C*. *haemulonii* (except 1, as *C*. *catenulata*) by Phoenix (BD-Diagnostics, Sparks, MD), as *C*. *haemulonii* by VITEK, and as *C*. *famata*, *C*. *lusitaniae*, *C*. *guilliermondii*, or *C*. *parapsilosis* by MicroScan (Beckman Coulter, Pasadena, CA) [25] (Table 1). However, Matrix-assisted laser desorption ionization–time of flight mass spectrometry (MALDI-TOF MS) is considered a more rapid and robust diagnostic technique for *C*. *auris* identification [9, 10, 13, 16]. Currently, the MALDI-TOF MS approach is commercialized by mainly 2 manufacturers, namely MALDI Biotyper (Bruker-Daltonics, Bremen, Germany) and VITEK MS (BioMérieux). The MALDI Biotyper (Bruker-Daltonics) has a database library that contains spectra of 3 strains of *C*. *auris*: 2 from Korea and 1 from Japan. Although both the Bruker-Biotyper and VITEK-MS MALDI-TOF systems lack *C*. *auris* entries in the FDA-approved libraries, the research-use-only libraries contain the *C*. *auris* database in both MALDI-TOF MS systems [25]. Due to the fact that this yeast is MDR, it is important to identify these species correctly in order to provide optimal patient care.

**Table 1:** Worldwide reports of *Candida auris* along with their misidentification using commercial systems and frequency of antifungal resistance.

Country	Number of *Candida auris* isolates	Sample (number)	Biochemical misidentification (System)	Molecular/MALDI-TOF MS identification	Number of isolates					Year of publication [References]
					FLU (≥32 μg/ml)	ITC (≥1 μg/ml)	VRC (≥2 μg/ml)	Echinocandins (≥8 μg/ml)	AMB (>1 μg/ml)	
**Japan**	1	Ear discharge	ND	ITS, D1D2	ND	ND	ND	ND	ND	2009 [23]
**Korea**	15	Ear discharge	ND	ITS, D1D2	8	8	2	none	9	2009 [2]
**South Korea**	6	Blood	*C*. *haemulonii* (VITEK), *Rhodotorula glutinis* (API20C-AUX)	ITS, D1D2	4	2	none	none	3	2011 [3]
**India**	12	Blood	*C*. *haemulonii*, *C*. *famata* (VITEK); *C*. *sake* (API20C-AUX)	ITS, D1D2	10	none	none	none	none	2013 [4]
	15	Blood (7), CVC tip (3), Excised tissue (3), BAL (1), pus (1)	*C*. *haemulonii* (VITEK)	ITS	15	none	7	none	none	2014 [5]
	4	Blood (1), urine (1), pericardial fluid (1), BAL (1)	*C*. *haemulonii* (VITEK); *C*. *sake* (API20C-AUX)	ITS, D1D2	4	none	none	none	none	2014 [6]
	102	Blood (78), tissue (4), pleural fluid (6), peritoneal fluid (7), urine (4), sputum (3)	*C*. *haemulonii/C*. *famata* (VITEK)	ITS, MALDI-TOF MS	80	none	32	none	14	2015 [9]
	51	Blood	Not mentioned	ITS, D1D2	49	3	9	none	10	2017 [19]
**India, South Africa, Korea, Japan, Brazil**	104: 90 India (I), 6 South Africa (SA), 5 Brazil (B), 2 Korea (K), 1 Japan (J)	Blood (*n* = 89; 78 I, 6 SA, 5 B), peritoneal and pleural fluid (5), invasive infections (4), urine (1), sputum (2)	*C*. *haemulonii* (VITEK)	ITS, D1D2, MALDI-TOF MS	5 (SA); 5 (B); 1 (K); none (J)	None (SA); none (B); 1 (K); none (J)	1 (SA); 5 (B); 1 (K); none (J)	none (SA); none (B); none (K); none (J)	none (SA); 3 (B); none (K); none (J)	2016 [10]^a^
**Kuwait**	1	Blood	*C*. *haemulonii* (VITEK)	ITS, D1D2	1	ND	none	none	none	2015 [8]
**Israel**	6	Blood (5), urine (1)	*C*. *haemulonii* (VITEK)	ITS, D1D2	6	none	none	none	6	2017 [18]
**Spain**	8	Blood (4), catheter tip (4)	*Saccharomyces cerevisiae* (AuxaColor 2); *C*. *sake* (API20C-AUX); *C*. *lusitaniae*, *C*. *haemulonii* (VITEK)	ITS	8	none	8	none	none	2017 [17]
**UK**	12	Blood, sputum, CSF, pleural fluid, arterial line, pustule swab, wound swab, femoral line		ITS, D1D2, MALDI-TOF MS	5	ND	1	none	none	2016 [11]
	50	Blood (16), wound (3), urinary catheter (1), unknown site with invasive candidiasis (2), colonization (28)^b^		MALDI-TOF MS	50	ND	ND	none	Range 0.5–2 μg/ml	2016 [13]
**Kenya**	21	Blood	*C*. *haemulonii* (VITEK)	ITS	-	-	-	-	-	2014 [24]
**South Africa**	4	Blood	*C*. *haemulonii* (VITEK) and *R*. *glutinis* (API20C-AUX)	ITS, D1D2	4	none	1	none	none	2014 [7]
**US**	7	Blood (5), urine (1), external ear canal (1)		Whole genome sequencing	5^c^	ND	ND	1^c^	1^c^	2016 [14]
**CDC** Collaborative Project **[Pakistan (n = 18), India (n = 19), South Africa, (n = 10), Venezuela (n = 5), Japan (n = 1)]**	54	Blood (27), urine (10), soft tissue (5), other sites (12)		D1D2, Whole genome sequencing	50	Range 0.125–2 **μ**g/ml	29	4	19	2017 [15]
**US**	10	NA	*R*. *glutinis* (API20C-AUX); *C*. *haemulonii*, *C*. *catenulata* (BD Phoenix); *C*. *haemulonii* (VITEK); *C*. *famata*, *C*. *lusitaniae*, *C*. *guilliermondii*, or *C*. *parapsilosis* (MicroScan)	ITS, D1D2, MALDI-TOF MS	ND	ND	ND	ND	ND	2017 [25]
**US,** tested strains from **Germany (*n* = 2), India (*n* = 11), Korea (*n* = 2), Japan (*n* = 1)**	16	Blood (15), ear (1)	Unidentified (API20C-AUX)	ITS	8	5	5^d^	none	12^e^, 16^d^	2017 [20]
**Venezuela**	18	Blood	*C*. *haemulonii* (VITEK)	ITS	18	ND	18	none	Range 1–2 **μ**g/ml	2016 [12]
**Colombia**	17	Blood (13) peritoneal fluid (1), CSF (1), bone (1), urine (1)	*C*. *haemulonii* (VITEK, Phoenix); *C*. *tropicalis* (MicroScan Walkaway); *C*. *famata* (API Candida)*; C*. *albicans* (MicroScanautoSCAN); *C*. *tropicalis* (MicroScan Walkaway)/ *C*. *famata* (API Candida); *C*. *albicans* (MicroScanAutoSCAN)	MALDI-TOF MS	10	ND	4	none	11	2017 [16]

Abbreviations: -, not clear in the abstract; AMB, amphotericin B; BAL, bronchoalveolar lavage; CDC, US Centers for Disease Control and Prevention; CSF, cerebral spinal fluid; CVC tip, central venous catheter tip; FLU, fluconazole; ITC, itraconazole; ITS, internal transcribed spacer; MALDI-TOF MS, Matrix- assisted laser desorption ionization–time of flight mass spectrometry; MIC, minimum inhibitory concentration; ND, not done; VRC, voriconazole.

^a^Antifungal susceptibility testing data of Indian isolates is same as reported by Kathuria et al., 2015.

^b^ Colonization with *C*. *auris* was defined as culture-positive skin, oropharynx, vascular line exit site, respiratory, and urinary tract without clinical signs of *Candida* infection.

^c^MIC value not given.

^d^MICs read after 48 hours.

^e^MICs read after 24 hours.

## Does genetic predisposition make *C*. *auris* virulent?

A recently published draft genome of *C*. *auris* shows that it has a genome size of approximately 12.3 Mb [26, 27]. A significant percentage of genes in *C*. *auris* are devoted to central metabolism, a property that is common to pathogenic *Candida* and crucial for adaptation to divergent environments. In addition, *C*. *auris* shares numerous virulence attributes with *C*. *albicans*, including genes and pathways involved in cell wall modelling and nutrient acquisition, histidine kinase-2 component systems, iron acquisition, tissue invasion, enzyme secretion, and multidrug efflux [21, 26, 27]. However, in vitro results in a single study that tested the production of phospholipase and secreted proteinase in multiple isolates of *C*. *auris* from different geographical regions showed that both secreted proteinase and phospholipase production was strain dependent. The phospholipase activity and secreted proteinase were detected in 37.5% and 64% of the tested isolates, respectively [20]. In general, the tested *C*. *auris* strains tended to have weak phospholipase activity, with the majority of isolates being non-phospholipase producers [20]. Furthermore, a significant portion of the *C*. *auris* genome encodes the ATP-binding cassette (ABC) and major facilitator superfamily (MFS) transporter families along with drug transporters that may explain the exceptional multidrug resistance in this pathogen [21, 27]. ABC-type efflux activity by Rhodamine 6G transport was significantly greater among *C*. *auris* than *C*. *glabrata* isolates, suggesting the intrinsic resistance of *C*. *auris* to azoles [18].

Interestingly, comparison of whole genome sequencing (WGS) data shows *C*. *auris* to be a close phylogenetic relative of *C*. *lusitaniae*, a species recognized for intrinsic antifungal resistance [21, 27]. *C*. *auris* also demonstrates thermotolerance, growing optimally at 37°C and maintaining viability at up to 42°C, salt tolerance, and cell aggregation into large, difficult-to-disperse clusters, which may help some strains to persist in the hospital environment [11, 23]. In a *Galleria mellonella* model, the aggregate-forming isolates exhibit significantly less pathogenicity than their non-aggregating counterparts [11]. Importantly, the non-aggregating isolates exhibited pathogenicity comparable to that of *C*. *albicans*, which is the most pathogenic member of the genus [11]. However, it is important to mention here that the observations made in this study are yet to be correlated with clinical cases and thus, assuming the same results in patients, need further experimentation. Furthermore, the virulence of *C*. *auris* tested in a mouse model of hematogenous-disseminated candidiasis showed distinct yeast cell aggregates in the kidneys of mice, with lethal *C*. *auris* infection suggesting that aggregation might be a mode of immune evasion and persistence in tissue [18]. Another significant factor involved in *C*. *auris* virulence is its ability to differentially adhere to polymeric surfaces, form biofilms, and resist antifungal agents that are active against its planktonic counterparts [28]. However, a more recent study reported that *C*. *auris* biofilms were significantly thinner, i.e., exhibited 50% thickness compared to *C*. *albicans* biofilm [20]. Also, *C*. *auris* exhibits minimal ability to adhere to silicone elastomer (a representative catheter material) relative to *C*. *albicans* [20]. *C*. *auris’*s weak adherence ability suggests that it is likely to play some role in catheter-associated candidiasis but not a large one, in contrast to *C*. *albicans* and *C*. *parapsilosis*, which are known to cause such infections [20]. Although, *C*. *auris* expresses several virulence factors, albeit to a lesser extent than *C*. *albicans* and in a strain-dependent manner [20].

## The past and present of *C*. *auris*: Is the emergence of *C*. *auris* a menace to public health?

In 2009, 15 isolates of *C*. *auris* were recovered from the ear canals of patients suffering from chronic otitis media in South Korea [2]. Most of these isolates showed a reduced susceptibility to AMB and azole antifungals. This report was followed by the first 3 cases of nosocomial fungemia caused by *C*. *auris* from South Korea [3]. The latter study reported that the earliest isolate of *C*. *auris* was found in 1996 in the Korean isolate collection [3]. All 3 patients had persistent fungemia for 10 to 31 days, and 2 patients who received FLU therapy followed by AMB showed therapeutic failure and had fatal outcome. Subsequently, 2 larger series of candidemia and deep-seated infections from India in 2013 and 2014 clearly showed that clonal strains of MDR *C*. *auris* had emerged in 3 hospitals [4, 5]. The isolates were resistant to FLU and 5-flucytosine (FC) and had elevated minimum inhibitory concentrations (MICs) of voriconazole (VRC) and caspofungin (CFG) [4, 5]. The most worrisome findings were persistent candidemia and high attributable mortality rates [4, 5]. *C*. *auris* accounted for >5% of candidemia in a national ICUs survey and up to 30% of candidemia at individual hospitals in India [4, 19]. In the subsequent 2 years, several reports of hospital-associated infections emerged from South Africa, United Kingdom, Venezuela, Colombia, United States, Pakistan, Israel, Kenya, and Spain [7, 11–18, 24]. Table 1 lists several countries reporting *C*. *auris* infection published so far across 5 continents. A collaborative project undertaken by the US Centers for Disease Control and Prevention (CDC) to understand the global emergence and epidemiology of *C*. *auris* reported that isolates from 54 patients with *C*. *auris* infection from Pakistan, India, South Africa, and Venezuela showed that 93% of isolates were resistant to FLU, 35% to AMB, and 7% to echinocandins; 41% were resistant to 2 antifungal classes, and 4% were resistant to 3 classes [15]. The fact that this yeast exhibits MDR clonal strains that are nosocomially transmitted is unusual in other *Candida* species [3, 5, 21]. Therefore, the possible threat of rapid spread in affected countries and its emergence in unaffected countries will not only challenge clinicians for its effective therapeutic management but will also bring high economic burden, especially to countries in resource-limited settings where modern identification facilities and access to antifungals other than FLU are limited.

## What are the drivers of clonal transmission and nosocomial outbreaks of *C*. *auris*?

There is increasing evidence that suggests likely transmission of *C*. *auris* in healthcare settings. Recent reports highlight the persistent colonization by *C*. *auris* of hospital environments and multiple body-sites of patients, leading to high transmissibility and protracted outbreaks [13, 14]. A large outbreak of 50 *C*. *auris* cases in a London cardio-thoracic center between April 2015 and July 2016 showed persistent presence of the yeast around bed-space areas [13]. Genotyping with amplified fragment length polymorphism (AFLP) demonstrated that *C*. *auris* isolates clustered. Similarly, the investigation of the first 7 cases of *C*. *auris* infection identified in the US, which occurred between May 2013 and August 2016, showed colonization with *C*. *auris* on skin and other body sites weeks to months after their initial infection, which could possibly lead to contamination of the healthcare environment and pose a risk of continuous transmission [14]. Furthermore, *C*. *auris* was isolated from samples taken from the mattress, bedside table, bed rail, chair, and windowsill [14]. WGS results demonstrate that isolates from patients admitted to the same hospital in New Jersey were nearly identical, as were isolates from patients admitted to the same Illinois hospital [14]. Also, in the London outbreak, a healthcare worker caring for a heavily *C*. *auris*–colonized patient had a *C*. *auris*–positive nose swab [13]. Effective implementation of strict infection-prevention control measures are required to prevent transmission of *C*. *auris*. These include isolation of patients and their contacts, wearing of personal protective clothing by healthcare workers, screening of patients on affected wards, skin decontamination with chlorhexidine, environmental cleaning with chlorine-based reagents, and terminal decontamination with hydrogen peroxide vapor or ultraviolet (UV) light [13, 29]. Enhanced terminal cleaning with UV light has recently been shown to reduce infections with many nosocomial pathogens and might also be of use for preventing *C*. *auris* transmission [30].

Previously, several geographically related clusters have been reported from South Korea [2, 3], India [4, 5, 10], South Africa [10], Pakistan [15], and hospitals in Latin America [12, 16]. Clonality within *C*. *auris* has been shown using AFLP, multilocus sequence typing, and MALDI-TOF MS among strains in India, South Africa, and Brazil [10]. A recent study applying WGS demonstrated highly related *C*. *auris* isolates in 4 unrelated and geographically separated Indian hospitals, suggesting that this pathogen exhibits a low diversity [21]. A large-scale application of WGS analysis suggests recent independent and nearly simultaneous emergence of different clonal populations on 3 continents, demonstrating highly related *C*. *auris* isolates in the same geographic areas [15]. So far, no reservoir of *C*. *auris* has been identified, although future studies on its isolation from animals, plants, and water sources are warranted.

## Is antifungal resistance in *C*. *auris* a therapeutic challenge?

Patients with *C*. *auris* infections have risk factors similar to those of other *Candida* spp. infections, including abdominal surgery (25%–77%), broad-spectrum antibiotics (25%–100%), ICU admission (58%), diabetes mellitus (18%), presence of central venous catheters (25%–94%), and malignancies (11%–43%) [3–5, 7, 12, 14–16]. The overall crude in-hospital mortality rate of *C*. *auris* candidemia ranges from 30% to 60%, and infections typically occur several weeks (10‒50 days) after admission [4, 5, 10, 12, 13]. *C*. *auris* invasive infections represent a therapeutic challenge, and no consensus exists for optimal treatment. A few studies report breakthrough fungemia while on FLU, and this correlates with commonly reported high MICs (>32 **μ**g/ml), suggesting intrinsic resistance against this drug [3–5]. Although epidemiological cutoff values (ECVs) or clinical breakpoints are not yet defined for *C*. *auris*, newer azoles such as posaconazole (range, 0.06–1 **μ**g/ml) and isavuconazole (range, <0.015–0.5 **μ**g/ml) show excellent in vitro activity against *C*. *auris* [4, 5, 7, 15, 19]. Analysis of antifungal data published in various studies and depicted in Table 1 clearly shows that about 90% of strains tested are resistant to FLU. Regarding VRC, elevated MICs are reported in 50% of isolates in 2 large series published from India and the CDC [9, 15]. Furthermore, variable susceptibility has been seen with AMB: 15%–30% of the isolates exhibit high (>2 **μ**g/ml) MICs [9, 15]. Up till now, echinocandin resistance is noted in fewer isolates (2%–8%) [9, 14, 15], but almost half of isolates are MDR (resistant to ≥2 antifungal classes), and a low number (4%) exhibit resistance to all classes of antifungals [2, 9, 12, 15, 16, 19]. Echinocandins remain the first-line therapy for *C*. *auris* infections, provided that specific susceptibility testing is undertaken at the earliest opportunity. Although CFG is normally highly effective against *Candida* biofilms, a recent report demonstrated that CFG was predominately inactive against *C*. *auris* biofilms [29]. FC (MIC_50_, 0.125–1 **μ**g/ml) is a treatment option in renal tract or urinary tract infections, as the echinocandins fail to achieve therapeutic concentrations in urine [4, 5, 7, 9, 11–13, 15, 18]. Also, a novel drug, SCY-078, which is the first orally bioavailable 1, 3-β-D-glucan synthesis inhibitor, has been shown to possess potent activity against various *Candida* spp. and exhibit potent antifungal activity against *C*. *auris* isolates [20]. Furthermore, SCY-078 showed growth-inhibition and anti-biofilm activity and could be an important antifungal to treat this MDR species [20]. At present, the mechanism of antifungal resistance in *C*. *auris* is unclear. The recently published draft genome of *C*. *auris* revealed the presence of single copies of *ERG3*, *ERG11*, *FKS1*, *FKS2*, and *FKS3* genes [21]. Detection of azole-resistant mutations by comparing *ERG11* amino acid sequences between *C*. *albicans* and *C*. *auris* showed that alterations at azole-resistance codons in *C*. *albicans* were present in *C*. *auris* isolates [15]. These substitutions were strongly associated with country-wise–specific geographic clades [15]. Resistance is probably inducible under antifungal pressure, resulting in rapid mutational changes. However, future studies with emphasis on several molecular mechanisms, including efflux and transporters, could provide insight on *C*. *auris* resistance.

## What are the important things that we still need to learn about *C*. *auris*?

We are just beginning to know the epidemiology and behavior of *C*. *auris*, but at the present, far more gaps exist in our knowledge. The earliest findings of *C*. *auris* are from 1996. The pertinent question remains whether this pathogen existed far earlier than 1996, and we were just unable to identify it. The latter is less plausible because many centers have reviewed archived isolate collections that have not shown any isolates of *C*. *auris* before 1996. We also do not know why *C*. *auris* is independently, almost simultaneously, emerging in so many places worldwide. It has been shown that there is a profound phylo-geographic structure with large genetic differences among geographic clades and high clonality within the geographic clades. However, a common characteristic is the high level of antifungal resistance, which is rare in other *Candida* spp. *C*. *auris* is the only species in which several isolates have been identified with resistance to all 4 classes of human antifungal drugs. It seems reasonable to opine that changes or misuse of antifungal drugs is one of the factors, although no specific risk factors for acquiring *C*. *auris* seem to exist. What we do know is that environmental factors probably play a role in outbreaks in healthcare settings that include prolonged survival in healthcare environments, probably due to skin colonization of patients and asymptomatic carriers. It is obvious that future research is warranted on multiple aspects of *C*. *auris*, which seems to have the typical characteristics of well-known, healthcare-associated pathogens such as carbapenemase-producing gram-negatives, *Clostridium difficile*, vancomycin-resistant *Enterococcus* (VRE), and methicillin-resistant *Staphylococcus aureus* (MRSA). Given the behavior of the latter 4, a further spread of *C*. *auris* in healthcare settings on a worldwide scale is expected. *C*. *auris* worldwide emergence has prompted the CDC, (http://www.cdc.gov/fungal/diseases/candidiasis/candida-auris-alert.html [last accessed February 2017]), Public Health England (PHE), London (https://www.gov.uk/government/uploads/system/uploads/attachment_data/file/534174/Guidance_Candida__auris.pdf [last accessed February 2017]), and the European Centre for Disease Prevention and Control (ECDC), Europe (http://ecdc.europa.eu/en/publications/Publications/Candida-in-healthcare-settings_19-Dec-2016.pdf) to issue health alerts for strict vigilance of *C*. *auris* cases. International collaborative consortia and timely efforts by the medical community are indispensable in controlling this super bug before it adapts in our healthcare facilities. Furthermore, more intensive efforts are required, and one such crucial step is the support from funding agencies to initiate multidisciplinary research to better understand its ecology, evolution, and resistance mechanisms, which will go a long way for its treatment and prevention.
